# Effect of Cell Thickness on the Electro-optic Response of Polymer Stabilized Cholesteric Liquid Crystals with Negative Dielectric Anisotropy

**DOI:** 10.3390/ma13030746

**Published:** 2020-02-06

**Authors:** Kyung Min Lee, Ecklin P. Crenshaw, Mariacristina Rumi, Timothy J. White, Timothy J. Bunning, Michael E. McConney

**Affiliations:** 1 Air Force Research Laboratory, Materials and Manufacturing Directorate, Wright-Patterson Air Force Base, OH 45433, USA; ecklin.crenshaw.ctr@us.af.mil (E.P.C.); Mariacristina.Rumi.1.ctr@us.af.mil (M.R.); timothy.bunning@us.af.mil (T.J.B.); 2 Azimuth Corporation, Beavercreek, OH 45431, USA; 3 Department of Chemical and Biological Engineering, University of Colorado, Boulder, CO 80309, USA; timothy.j.white@colorado.edu

**Keywords:** cholesteric liquid crystals, red tuning, switching responses, polymer stabilization

## Abstract

It has previously been shown that for polymer-stabilized cholesteric liquid crystals (PSCLCs) with negative dielectric anisotropy, the position and bandwidth of the selective reflection notch can be controlled by a direct-current (DC) electric field. The field-induced deformation of the polymer network that stabilizes the devices is mediated by ionic charges trapped in or near the polymer. A unique and reversible electro-optic response is reported here for relatively thin films (≤5 μm). Increasing the DC field strength redshifts the reflection notch to longer wavelength until the reflection disappears at high DC fields. The extent of the tuning range is dependent on the cell thickness. The transition from the reflective to the clear state is due to the electrically controlled, chirped pitch across the small cell gap and not to the field-induced reorientation of the liquid crystal molecules themselves. The transition is reversible. By adjusting the DC field strength, various reflection wavelengths can be addressed from either a different reflective (colored) state at 0 V or a transparent state at a high DC field. Relatively fast responses (~50 ms rise times and ~200 ms fall times) are observed for these thin PSCLCs.

## 1. Introduction

The properties of composite materials containing liquid crystalline and polymeric components vary widely depending on the relative content of the low and high-molecular-weight fractions and the nature of the constituents [1,2]. Polymer-stabilized liquid crystals (PSLCs) are a class of such composites and typically consist of a low-molecular-weight liquid crystal (LC) and a polymer formed from the in-situ polymerization of a reactive mesogen mixed with the LC component [2,3,4]. The polymerization is carried out while the system is in the LC ordered phase and the initial alignment of the reactive mesogen is retained in the resulting polymer chains. The polymer fibrils thus formed stabilize the liquid crystal by functioning as alignment surfaces distributed throughout the liquid crystalline phase. When the LC is initially in the cholesteric phase, the composites are called polymer-stabilized cholesteric liquid crystals (PSCLCs). In both PSLCs and PSCLCs, the polymer content is generally low (typically below 10%) and, although the composites exhibit temperature and electro-optic responses, these differ from those of the bulk LCs on which they are based [2,4,5,6]. For example, an increase in temperature in a PSLC leads to a sharp decrease in the order parameter near the clearing temperature of the LC [7,8]. However, complete isotropization is not achieved even well above the clearing temperature, because the polymer network and a fraction of LC molecules in the immediate vicinity of the network remain ordered at all temperatures. In PSLCs and PSCLCs containing a cholesteric liquid crystal (CLC) with positive dielectric anisotropy, the low-molecular-weight CLC molecules can be reoriented by an external electric field if the polymer content is not too high, although threshold voltages are larger than in bulk LC [1,5,7,9,10,11]. The recovery to the equilibrium state, however, is typically faster in polymer-stabilized systems, independent of the cell thickness, because of the restoring force exerted by the polymer network. 

The polymer network in a PSCLC can also be used as a scaffold to order and align low-molecular-weight molecules other than those present during its fabrication: the initial CLC molecules can be leached out of the composite (for example, by immersion in an appropriate solvent), leaving behind the insoluble ordered, polymeric fraction. After removal of the solvent, the system can be refilled with another liquid crystal [1,2,12]. The templating effect exerted by the ordered polymer network can be strong enough to align non-mesogenic molecules or to overcome the initial handedness of a chiral LC (e.g., when the template is refilled with a CLC of the opposite handedness, the CLC fluid takes on the handedness of the template). Structurally chiral scaffolds can template the CLC phase even if they are not derived from mesogenic precursors [13]. This washout/refill method has been used to obtain polymer/LC composites with unique properties not exhibited by natural systems. For example, it allowed the fabrication of hyper-reflective devices, in which both right-handed and left-handed circularly polarized light is reflected from a single cell [12,14,15,16]. 

In traditional CLCs, the pitch P_0_ of the CLC structure, the distance along the helical axis over which the director orientation changes by 2π, is inversely proportional to the concentration of the added chiral dopant. The selective Bragg reflection due to the 1D periodicity of the refractive index is observed as a notch centered at

λ_0_ = n_avg_ P_0_ (1)

with reflection bandwidth Δλ = Δn P_0_, where n_avg_ = (n_o_ + n_e_)/2 is an average refractive index of the liquid crystal (n_o_ and n_e_ are the ordinary and extraordinary refractive indices, respectively) and Δn = n_e_ − n_o_ is the birefringence [1,17]. These expressions are applicable to normal incidence. In PSCLCs, the position and bandwidth of the Bragg reflection become decoupled from the properties of the low-molecular-weight LC component. Typically, the pitch P_0_ of the CLC before polymerization is locked during the polymerization. However, n_avg_ and Δn in a PSCLC can be different from those of the starting system when the structure has been refilled with a new LC or liquid. Additionally, effective bandwidths larger than Δn P_0_ can be achieved if a gradient of pitch values can be generated in the system [18,19,20,21].

**Figure 1:**
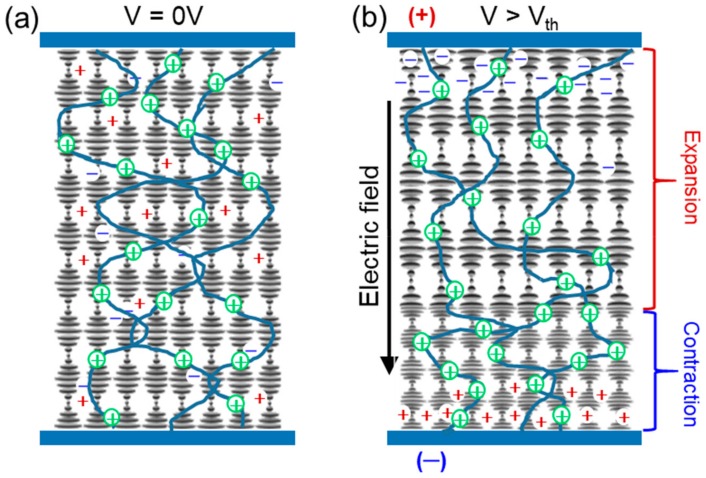
Schematic of the polymer network in a deformable polymer-stabilized cholesteric liquid crystal (PSCLC) with Δε < 0: (**a**) with no applied field, (**b**) with a DC field applied between the top and bottom substrates. The blue lines represent the polymer network, the grey horizontal bars are the low-molecular-weight cholesteric liquid crystal (CLC) molecules, + and – are the free cationic and anionic impurities and ⊕ are trapped cationic impurities.

If the low-molecular-weight component of the PSCLC has a negative dielectric anisotropy (Δε < 0), electro-optic responses very different from those of non-stabilized CLCs can be induced. When a voltage is applied across the cell in a Δε < 0 PSCLC with planar alignment (so that the electric field is parallel to the helical axis), there is no direct field-induced reorientation as the molecules are already oriented perpendicular to the field and experience no electric-field induced torque. However, it has been shown that the application of a direct-current (DC) field can produce broadening or tuning of the reflection notch [22,23]. These PSCLC systems return to their equilibrium conditions when the field is removed. PSCLCs with Δε < 0 have been studied extensively in this laboratory over the past several years [22,23,24,25,26,27,28]. The current understanding of the responsible mechanism [25,27,28] is based on the movement of ions present in the composite, which drift toward the oppositely charged electrodes (red and blue charges in Figure 1) when a DC field is applied. These ions, present in densities on the order of 10^9^–10^14^ ions/cm^3^, are residual impurities (initiators, catalysts, salts, moisture) originated in the synthesis and purification processes [29]. A fraction of the positively charged ions are not free but are trapped on or near the polymer network (green charges in Figure 1a). As these trapped ions drift in the electric field, the polymer fibrils to which they are attached (blue lines in the figure) are dragged along. This leads to the physical deformation of the polymer network and a change in the local period of the structure across the cell gap, as can be seen in Figure 1b. The deformation continues until elastic forces that tend to restore the polymer to its undeformed state counterbalance the electrostatic force on the ions. Due to the anchoring to the polymer network, the low-molecular-weight CLC molecules reorient to adjust to the new local order (grey bars in the figure). During the deformation, the length of the polymer network does not change if the network is attached to the substrates, and thus the total number of repeat units (half pitches) over the cell thickness is constant. However, the pitch is no longer uniform, being contracted in some regions (on the negative electrode side) and expanded in others (on the positive electrode side). The details of the field-induced deformation and of the pitch distribution is expected to depend on the nature of the liquid crystal, the polymer, and the impurities, and on the chemical and physical characteristics of the crosslinked network.

**Figure 2:**
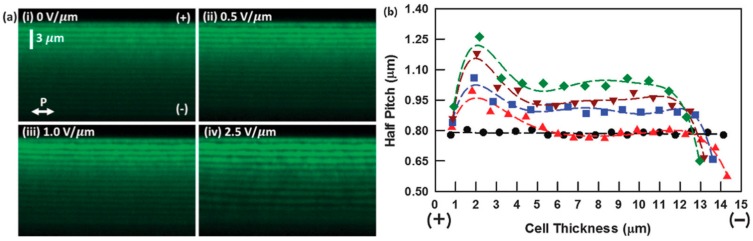
(**a**) Map of the fluorescence intensity by three-photon excitation along a 2D cross-section of a PSCLC without or with an applied electric field (reflection notch at 2.45 μm at zero field and ~15 μm cell thickness). The vertical direction is along the cell normal and the helical axis, the horizontal direction is parallel to the cell substrates. The white arrow labeled ‘‘P’’ in (i) is the polarization direction of the excitation light. The polarity of the DC voltage is indicated by the “(+)” and “(−)“ signs. (**b**) Magnitude of the half-pitch through the cell thickness as obtained from the fluorescence images in (**a**) for the following values of the electric field: (●) 0 V μm^−^^1^, (▲) 0.5 V μm^−^^1^, (■) 1.0 V μm^−^^1^, (▼) 2.0 V μm^−^^1^, and (◆) 2.5 V μm^−^^1^. Composition of the CLC mixture: 0.2 wt% I-369, 4 wt% SL04151, 2 wt% RM23, 2 wt% CB15, and 91.8 wt% MLC 2079. The PSCLC was formed by curing the mixture with exposure to 365 nm UV light at 100 mW cm^−2^ for 5 min. Reproduced from Ref. [25] with permission from the Royal Society of Chemistry.

Confocal or multiphoton fluorescence polarization microscopy has been used to obtain three-dimensional maps of the director orientation in this class of PSCLCs [25,28]. It has been shown that the pitch undergoes a non-uniform distortion when a DC field is applied. In the example shown in Figure 2a [25], the period in the fluorescence intensity modulation starts to change near the top substrate when a DC field is applied and it increases with increasing field strength. The corresponding half pitches at various depths in the cell are shown in Figure 2b. It is seen that at low field (0.5 V μm^−1^) the pitch changes only near the top and bottom substrate, being contracted on one side and expanded on the other. At larger DC fields (≥1.0 V μm^−1^), the pitch deviates from the original value throughout the cell and, on average, increases with the field. For the sample in Figure 2 and similarly formulated ones, the Bragg reflection moves to longer wavelengths when the amplitude of the DC field is increased, but without a significant change in bandwidth [23,25].

Polymer deformation that yields a shift of the reflection notch toward shorter wavelengths is also possible, by careful selection of the precursors and polymerization conditions [26]. Other formulations of PSCLCs, instead, develop a pitch gradient in the presence of an electric field, which manifests spectroscopically as a broadening of the Bragg reflection around its initial position [22,27,28,30]. Deformation of the polymer network can also be induced in PSCLCs based on CLCs with Δε > 0 using DC electric fields [31]. In these latter devices, switching between a reflecting and a transparent state can be achieved through the application of alternating-current (AC) fields [31].

In this work, it is shown that switching between a reflective and a transparent state is possible in PSCLCs with Δε < 0 if the cell is kept thin (≤5 μm). The electro-optic response, studied for cells of different thicknesses, indicated that the tuning range decreased with decreasing thickness. Additionally, thin cells appear transparent at high fields because the number of repeat units of the deformed pitch that can be accommodated in the cell is too small to yield significant reflection efficiency. By adjusting the DC field strength, reflection peaks at various wavelengths and an optically clear state can be obtained. PSCLCs with stimuli-responsive optical properties, such as those reported here, can be potentially used in smart windows, optical components, and display applications.

## 2. Materials and Methods

### 2.1. Preparation of PSCLCs

Alignment cells were prepared from indium tin oxide-coated glass slides (Colorado Concept Coatings, Loveland, CO, USA). The glass substrates were cleaned in acetone and methanol and then treated with air plasma for several minutes. The substrates were spin-coated with a polyimide solution and baked on the hot plate at 200 °C for 30 min. The polyimide alignment layers were rubbed with a cloth, and the cell was constructed to ensure planar alignment conditions. The cell gap was controlled by mixing glass rod spacers (3–15 μm thickness) into an optical adhesive. The thickness of the cells was measured using an optical method based on the interference pattern of reflected light by the glass substrates in each empty cell [32]. The standard deviation in the thickness of each cell was measured to be less than 10%. Samples were prepared by mixing 0.4 wt% of the photoinitiator Irgacure 369 (BASF), two right-handed chiral dopants (R1011 and R811, Merck, Darmstadt, Germany), 6 wt% of a right-handed chiral LC monomer (SL04151, AlphaMicron, Inc., Kent, OH, USA), and a Δε < 0 nematic LC (MLC-2079, T_NI_ = 102 °C, Δε = −6.1, Δn = 0.15 at λ = 589 nm, Merck). The pitch length (reflection band position) of the CLC is adjusted by the concentration of the chiral dopants. For short pitch CLCs, a mixture of two right-handed chiral dopants was used to prevent phase separation of the chiral dopant from the LC mixture. The polymer stabilizing network was formed by photopolymerization with 100 mW cm^−2^ of 365 nm light (OmniCure LX500 LED Spot UV Curing System, Lumen Dynamics, Mississauga, ON, Canada) for 5 min. For these compositions and curing conditions, the resulting PSCLC samples exhibited electrically induced redshift of the reflection notch.

### 2.2. Experimental Setup and Measurements

Transmission spectra were collected with a fiber optic spectrometer (Ocean Optics, Dunedin, FL, USA). Unpolarized or right-handed circularly polarized light (RH CPL) was used as the probe beam. Transmission spectra were collected before, during, and after the application of DC fields, which were applied either progressively at the scanning rate of 1 V s^−1^ or directly in a single step. The dynamics of the electro-optic response was studied by monitoring the transmitted light over time with a fast photodiode and an oscilloscope. Rise and fall response times were estimated using a 90%/10% method.

## 3. Results and Discussion

**Figure 3:**
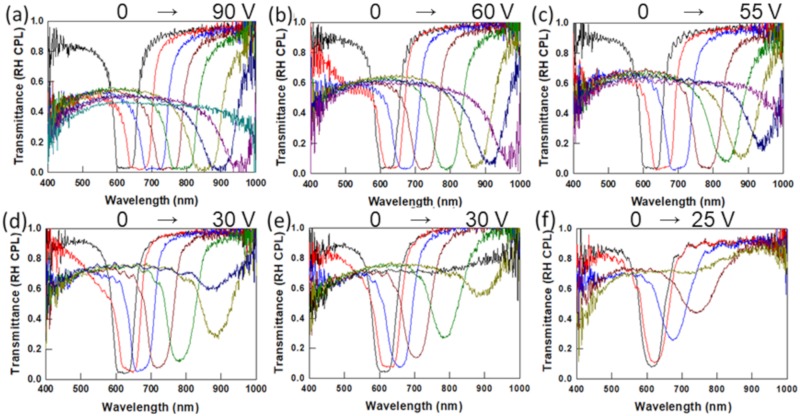
Transmission spectra of negative Δε PSCLCs with various cell thicknesses and DC voltages in the ranges shown above each graph: (**a**) 14.1 ± 0.3 μm thickness, 0 V to 90 V DC, (**b**) 10.3 ± 0.3 μm, 0 V to 60 V DC, (**c**) 7.6 ± 0.2 μm, 0 V to 55 V DC, (**d**) 4.9 ± 0.2 μm, 0 V to 30 V DC, (**e**) 3.6 ± 0.2 μm, 0 V to 30 V DC and (**f**) 2.5 ± 0.2 μm, 0 V to 25 V DC. Composition: 0.4 wt% I-369, 6 wt% SL04151, 5 wt% R1011, 4 wt% R811, and 84.6 wt% MCL 2079.

A series of cells with planar alignment was prepared with different thicknesses (from 2.5 to 14.1 μm). After filling the cells with the mixture described in Section 2, the polymer network was formed by photopolymerization under UV illumination. The pitch of the as-prepared PSCLCs was 0.40 μm. The pitch of the samples was quantified from Equation (1) using n_avg_ = 1.565. The transmission spectra were recorded as a function of the DC field applied between the top and bottom substrate (Figure 3). All PSCLCs exhibit electrically induced tuning of the reflection notch toward longer wavelengths, but the tuning range achievable is found to depend on the thickness of the cell. The 14.1 μm thick PSCLC shows a redshift of about 400 nm at 90 V DC (Figure 3a). The transmittance at the center of the reflection notch is very low (<5%) in this sample over the entire tuning range. The PSCLCs with thicknesses of 10.3 μm and 7.6 μm exhibit similar tuning ranges as the 14.1 μm cell, but the transmittance at the bandgap wavelength increases with increasing DC fields (Figure 3b,c). As discussed in Section 1, the redshift is a result of an electric-field induced deformation of the polymer network, yielding a non-uniform pitch distribution in the cell.

Thinner PSCLCs exhibit narrower tuning ranges and higher transmittance at the center of the reflection notch, as shown in Figure 3d–f. The 4.9 μm sample shows ~40% transmittance at about 900 nm for 25 V DC. It should be noted that right-handed circularly polarized light (RH CPL) was used as the probe beam. For a pitch of 0.40 μm, the number of repeat units (half pitches) in unbiased PSCLCs cells with 4.9 μm, 3.6 μm, and 2.5 μm thickness, are ~25, 18, and 13, respectively. In the biased samples, however, only a few repeat units are associated with a particular pitch in the deformed network and thus the reflection resulting from that portion of the network becomes less efficient. If the DC voltage is increased further, the PSCLC with 4.9 μm thickness becomes transparent (at 35 V DC). Similarly, the thinner PSCLCs with 3.6 μm and 2.5 μm thicknesses become transparent at 30 V DC and 25 V DC, respectively. This electric field-induced transparency occurs when the reflection efficiency from the deformed PSCLC becomes negligible because an insufficient number of repeat units for the extended pitch can be accommodated in the cell thickness.

**Figure 4:**
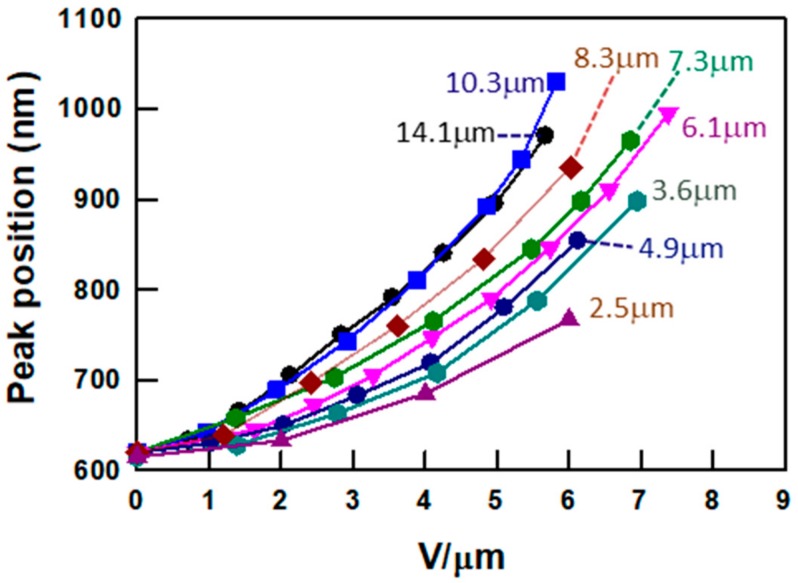
Peak position of the reflection band of PSCLCs with various cell thicknesses as a function of the DC field.

As mentioned above, the overall tuning range is narrower in the thinner cells. The reflection peak position plotted as a function of the DC field strength (V μm^−1^) is summarized in Figure 4. Previous measurements with thick samples indicate that the tuning is sensitive to electric field strength and independent of cell thickness. However, as shown in Figure 4, a strong thickness dependence is observed in the tuning at thicknesses below 10 μm. The broadening and distortion of the spectra in thinner cells at higher fields indicate that the thinner samples cannot fully accommodate the non-linear pitch deformation observed in the thicker cells. The tuning mechanism, as shown in Figure 1, involves an electrically induced contraction of the polymer network toward the negative electrode for some fraction of the cell thickness, which thereby stretches the remaining anchored fraction of the polymer network on the positive electrode side. In thicker samples, the stretched region has a fairly uniform pitch and therefore gives rise to a red-shifted reflection. In contrast, the contracted region has a highly chirped pitch and thus appears broad with little reflection efficiency. As the thickness of the cell decreases, the thickness associated with the stretched uniform pitch is reduced, so the range associated with red tuning decreases. As the field increases further beyond the red tuning range of the thin sample, the stretched region of the cell has also a non-uniform pitch distribution, which reduces the reflection efficiency and eventually at high fields the reflection notch is eliminated.

**Figure 5:**
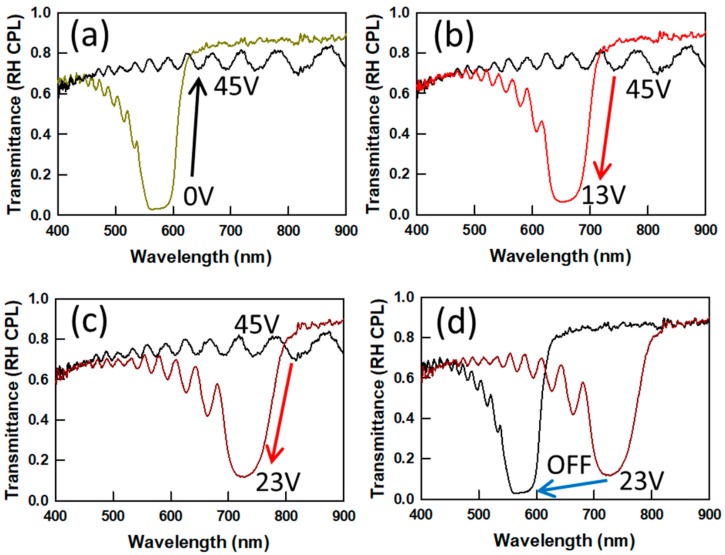
Transmission spectra of a Δε < 0PSCLC with 5 ± 0.2 μm thickness showing a (**a**) reflective to transparent or (**b**,**c**) transparent to reflective switching response by application of a DC voltage. (**d**) Spectra for the relaxation to the off state from 23 V.

**Figure 6:**
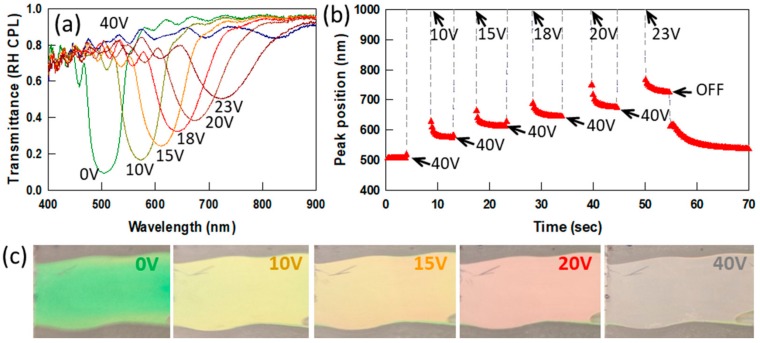
(**a**) Transmission spectra of a negative Δε PSCLC with 3 ± 0.2 μm thickness for DC voltages up to 40 V DC. (**b**) Peak position during the switching of the same PSCLC using various DC voltages. The arrows indicate the times at which the DC voltage was changed to the value indicated. (**c**) Photographs of the reflection color of the PSCLC as a function of applied DC voltage.

The loss of reflectance at high DC fields shown in Figure 3 can be exploited as a new switching mechanism for PSCLCs. Two examples are shown in Figure 5 and Figure 6 (an additional case is shown in Figure S1 of the Supplementary Materials). The case in Figure 5 with 5 μm thickness shows that direct switching from a reflective state at 575 nm to a transparent state (no reflection notch) is possible by the application of 45 V DC voltage (Figure 5a). When the voltage is reduced from 45 V DC to 13 V DC or 23 V DC, a reflection notch reappears, but at a shifted position, 670 nm or 730 nm, respectively (Figure 5b,c). When the applied DC voltage is removed, the reflection notch relaxes back to the original notch position (Figure 5d). By controlling the DC field, the spectral position of the reflection notch is thus tuned and switched off.

Figure 6 shows the red tuning and switching response of a 3 μm PSCLC cell. This PSCLC cell has an initial notch position of 500 nm (~9 pitches through the thickness). The reflection notch shifts to 730 nm at 23 V DC. The minimum transmittance of the unbiased sample is about 8%. As the DC voltage increases, the in-band transmittance increases due to the decrease in the number of repeat units with the elongated pitch, as discussed above. The reflection notch disappears at 40 V DC. When the voltage is reduced, the reflection notch reappears in a position determined by the magnitude of the voltage. This DC field controllable notch tuning and switching behavior is reversible (Figure 6b). Photographs of the PSCLC cell at various DC voltages are shown in Figure 6c, where the tunable reflection color is clearly visible.

**Figure 7:**
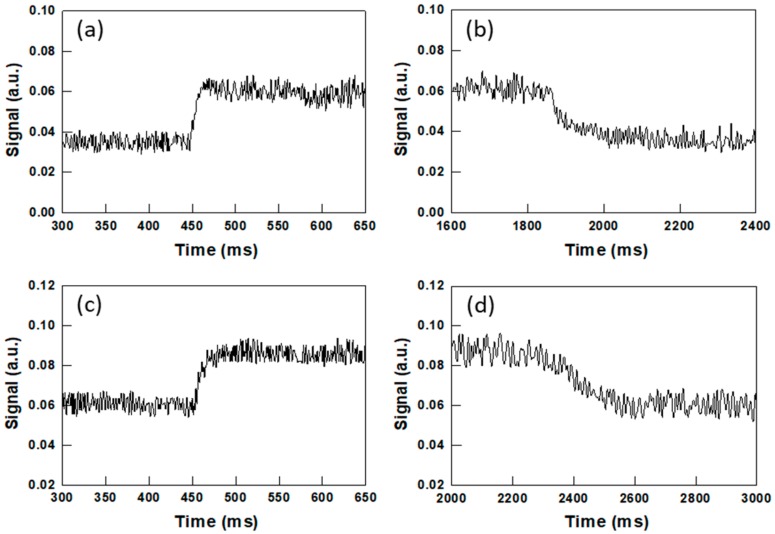
Time evolution of the intensity of the light transmitted by PSCLCs with (**a**,**b**) 3 ± 0.2 μm and (**c**,**d**) 5 ± 0.2 μm thickness when a DC voltage is switched on and off. For the PSCLC with 3 μm, a 40 V DC voltage is (**a**) applied directly at t ~ 450 and (**b**) removed at t ~ 1850 ms. Rise and fall times are ~30 ms and ~150 ms. For the PSCLC with 5 μm thickness, 45 V DC are (**c**) applied at t ~ 450 ms and (**d**) removed at t ~ 2300 ms. Rise and fall times are ~50 ms and ~200 ms.

The dynamics of the switching behavior in 3 μm and 5 μm PSCLCs cells is displayed in Figure 7. When the voltage is applied in one step (40 V DC for the 3 μm sample and 45 V DC for 5 μm thickness sample), there is a sudden change in transmittance that quickly levels off (Figure 7a,c). Using a 10%–90% method, the rise time for the 3 μm and 5 μm thickness samples are estimated to be ~30 ms and ~50 ms, respectively. When the voltage is switched off, the signal level decreases with fall times of ~150 ms and ~200 ms, respectively (Figure 7b,d). In contrast, a similar 15 μm PSCLC cell shows significantly slower responses, with rise and fall times in the 1.5–3 s range (Figure S2, Supplementary Materials) [33].

Additional work is required to better understand these effects and to develop improved correlations between the cell preparation conditions, the properties of the polymer network, and the electro-optical response of the PSCLCs. The switching mechanism reported here is different from the one reported in 2018 [31], where a DC electric field was used to deform the polymer network (as is the case for the tuning mechanism) and switching to a transparent state was achieved by the application of an AC field that induced the homeotropic orientation. In the present case, the transparent state is not homeotropic (no field-induced reorientation takes place in a Δε < 0 LC) and instead it has CLC order, albeit with negligible reflection efficiency, due to the pitch gradient and small thickness. The effect is also different from the bistable switching between a reflective and scattering state that was previously reported in other PSCLCs [34]. While the tuning range in these PSCLCs is smaller than in thick devices, it is still wide enough to be of interest in the design of variable color mirrors, with the additional feature that the reflection band can be switched off. Optimization of the material response for tuning range, switching time, or bandwidth can be addressed in follow-up investigations. The information thus developed can be used to evaluate the advantages and drawbacks of the proposed approach relative to other switching and tuning mechanisms in CLC-based materials for specific applications.

## 4. Conclusions

A study of the electro-optic response in PSCLC cells with Δε as been carried out as a function of cell thickness. In all samples, the application of a DC field leads to a redshift in the position of the reflection notch due to the field-induced deformation of the polymer network and a resulting chirped pitch, as previously reported. It is now shown that the tuning range of the reflection notch decreases with a decrease in the thickness of the sample. In addition, in thin cells, the reflection notch can be switched off if the DC field is increased above a critical value. The reflective state is restored when the field is removed. The switching times are on the order of 100 ms. This reflective-to-transparent “switching” in thin PSCLC samples is due to the fact that the number of effective repeat units in the deformed polymer network becomes too small to result in any measurable reflection efficiency, not to the reorientation of the low-molecular-weight LCs. The process is completely reversible. As the position of the reflection notch is field-dependent, the color of the reflective state can be dynamically controlled by applying an appropriate DC voltage. The combination of tuning and switching characteristics in a single device, as seen in this class of PSCLCs, could simplify the design of dynamic optical devices for various applications.

## Supplementary Materials

The following are available online at https://www.mdpi.com/1996-1944/13/3/746/s1, Figure S1: Transmission spectra of a negative Δε PSCLC with 5 μm thickness as a function of DC voltage. Figure S2: (a) Reflection band position and (b) response times of a PSCLC with 6% polymer content and ~15 μm thickness as a function of DC voltage. In (a) the voltage was turned on at t = 0 and off at t = 30 s. Data from Ref. [33].
