# Supplementary material for: Effect of Cell Thickness on the Electro-optic Response of Polymer Stabilized Cholesteric Liquid Crystals with Negative Dielectric Anisotropy
Source: Materials (Basel). 2020 Feb 6;13(3):746. doi: 10.3390/ma13030746 (PMC7040647; doi:10.3390/ma13030746)
Supplement: Supplementary file 1 [file materials-13-00746-s001.pdf]

Supplementary Materials:

Effect of Cell Thickness on the Electro-optic Response of Polymer Stabilized Cholesteric Liquid Crystals with Negative Dielectric Anisotropy

Kyung Min Lee [1,2,*], Ecklin P. Crenshaw [1,2], Mariacristina Rumi [1,2], Timothy J. White [3], Timothy J. Bunning [1] and Michael E. McConney [1,*]

1 Air Force Research Laboratory, Materials and Manufacturing Directorate, Wright-Patterson Air Force Base, OH 45433, USA; ecklin.crenshaw.ctr@us.af.mil (E.P.C.); Mariacristina.Rumi.1.ctr@us.af.mil (M.R.); timothy.bunning@us.af.mil (T.J.B.)

2 Azimuth Corporation, Beavercreek, OH 45431, USA

3 Department of Chemical and Biological Engineering, University of Colorado, Boulder, Boulder, CO 80309, USA; timothy.j.white@colorado.edu

* Correspondence: Michael.McConney.1@us.af.mil (M.E.M); Kyungmin.lee.3.ctr@us.af.mil (K.M.L.); Tel.: +1-937-255-9674 (M.E.M); +1-937-656-4695 (K.M.L.).

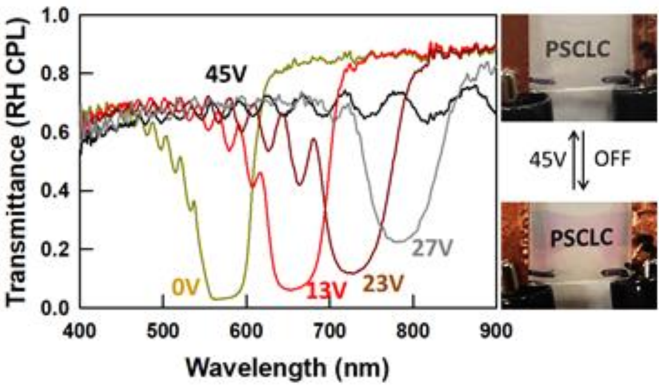

Figure S1. (Left) Transmission spectra of a negative Δε PSCLC with 5 ± 0.2 μm thickness as a function of DC voltage. The reflection notch shifts from 580 nm at 0 V to 790 nm at 27 V DC and disappears at 45 V DC. The initial pitch is ~0.39 μm and the cell thickness contains ~26 repeat units. (Right) At 0V the device is characterized by a pink transmission color (bottom image) and at 45V DC it is transparent (top image).

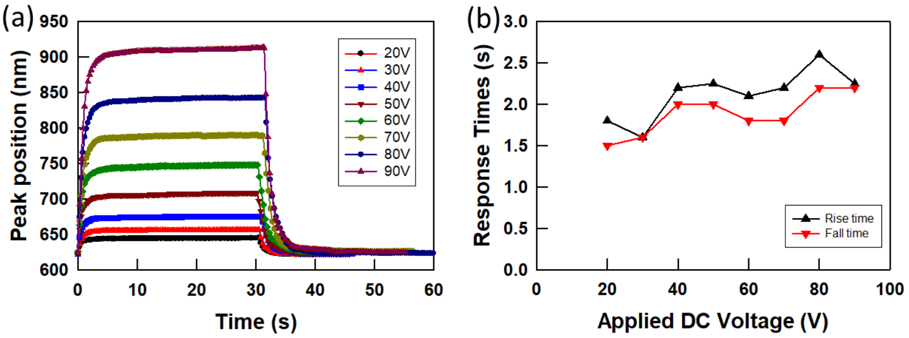

Figure S2. (**a**) Reflection band position and (**b**) response times of a PSCLC with 6% polymer content and ~15 μm thickness as a function of DC voltage. In (**a**) the voltage was turned on at t = 0 and off at t = 30 s. Data from Ref. [33].
